# 特殊人群的肺癌也需要我们大家关注

**DOI:** 10.3779/j.issn.1009-3419.2018.04.22

**Published:** 2018-04-20

**Authors:** 辉 李

**Affiliations:** 首都医科大学附属北京朝阳医院胸外科 Department of Thoracic Surgery, Beijing Chaoyang Hospital Affiliated to Capital Medical University

本文探讨了"HIV合并肺癌的个体化综合治疗"，是一篇非常有临床指导和参考价值的临床研究。

人类免疫缺陷病毒（human immunodeficiency virus, HIV）感染是对人群健康危害较大的传染病之一，它能引起人体免疫器官病变，造成免疫力低下，其最常表现为反应性增生和肿瘤性病变，例如卡波齐肉瘤和非霍奇金淋巴瘤等HIV相关性肿瘤。而肺癌则是最常见非HIV相关性肿瘤之一。但以往，由于AIDS生存期短、死亡率高，因此肺癌的发病率相对较低。随着高效抗逆转录病毒治疗（highly active anti-retroviral therapy, HAART）联合应用多种药物治疗使得HIV感染者的生存期明显延长，HIV相关性肿瘤的发生率也明显降低，然而随之而来的后果是其他恶性肿瘤最终呈现出发病率增加的趋势，肺癌也位居其中。因此，如何针对HIV合并肺癌患者进行治疗就成为一个重要的课题。上海市（复旦大学附属）公共卫生临床中心胸外科在这方面做了很多具有探索性和开创性的工作。本课题的研究结果必将对此类特殊人群的特殊疾病治疗提供重要的临床资料和经验。

HIV感染和AIDS目前仍是严重威胁人类健康的重大传染病。自1981年世界第一例HIV感染者被确诊以来，AIDS已造成全球3500多万人死亡。2017年，全球有100万人死于AIDS及相关病症。其中，非洲区域病情最严重，新发感染数约占全球的近三分之二。我国的HIV感染和AIDS虽不及非洲发病率高，但2010年至2016年总体呈上升趋势。据中国疾病预防控制中心性病艾滋病预防控制中心报告，2015年我国估计存活的艾滋病病毒感染者和艾滋病病人约占总人口的0.06%，截至2015年10月底，全国报告存活的艾滋病病毒感染者和病人共计57.5万例，死亡17.7万人。2017年1月至10月，中国AIDS发病数已达46, 206例，是2010年全年发病数的近3倍。值得关注的是，大约30%的感染者属于"隐形感染者"，即感染者自己不知道感染。这些"隐蔽"的感染者因为无法得到及时有效的治疗，疾病一直处在进展状态。与此同时，肺癌的发生风险也在增高。因此，我国的"抗艾"及"抗癌"的形势同样严峻。

研究证实，HIV感染是肺癌的一个独立的风险来源。一项来自美国的研究发现，在排除其他潜在风险（例如吸烟）后，HIV感染者患肺癌的发生率比非感染者高70%（HIV感染者：204/万人*vs* HIV非感染者119/万人）。肺癌正逐步成为HIV感染者致命的重要原因，这可能是由于HIV破坏了免疫系统所导致的。另一项研究发现，美国40%以上的艾滋病毒感染者吸烟，是普通人口的两倍以上。同时研究者认为，吸烟的AIDS死于肺癌的可能性比AIDS其他相关原因多了6倍-13倍。因此，肺癌已成为HIV感染者死亡的主要原因，肺癌的风险与艾滋病毒治疗的依从性有关。但戒烟可以大大降低患者死亡风险。肺癌的预防，特别是戒烟应该是AIDS患者综合治疗中的重要部分。

本研究的中期生存结果证实，HIV合并肺癌患者经不同的个体化综合治疗可以提高患者生存率，早期手术联合化疗效果显著。这也为今后的进一步研究和探索打下了坚实的基础。

